# Monthly Summary

**Published:** 1886-09

**Authors:** 


### Monthly Summary.

ALUMINIUM BRONZE IN MEDICINE AND DENTISTRY.—Professor C. Sauer, Instructor in Dentistry at the Royal University of Berlin, strongly recommends this bronze for the under layer of teeth-plates and other purposes. It is an alloy of nine parts of copper and one part of aluminium. It admits of almost as ready stamping and pressing as pure silver, (which next to pure gold, is the softest metal), and it has, besides, the elasticity of steel. In form of wire, aluminium bronze possesses a power of resisting tension approaching that of steel wire. These characteristics render it capable of substitution in many cases for silver, and for silver and gold alloys. The melting point of the bronze is higher than that of pure gold—1,000° C. It may, accordingly, be made red-hot without danger of melting, and manipulated with hard solder. It is soldered with fourteen or sixteen carat red gold, which is more capable of resisting chemical influences than silver solder, which contains zinc. Aluminium bronze is almost one-half lighter than fourteen carat gold.

Professor Sauer has made various experiments with aluminium alloys; the zinc alloy was unstable, the zinc oxidizing in the mouth, with gold and platinum alloys the aluminium decomposed, whereas in two cases aluminium bronze placed in the mouth under the influence of a galvanic current did not, after the lapse of four weeks, suffer loss of weight. The bronze oxidizes only superficially in the mouth. There forms upon it a kind of patina, such as is formed in the wearing of plates of fourteen, sixteen, eighteen, and even twenty carat gold. It admits of manufacture into spiral springs, plates, screws, canulas, &c., for surgical purposes. Even knives have been manufactured from it. A solution of corrosive sublimate of one to one thousand affects it superficially. For its disinfection carbolic acid is to be preferred, as it does not attack the bronze. Gold aluminium bronze acts similarly, but oxidizes to a greater extent, is softer, and not so elastic, and therefore is to be used as green gold or as twenty carat gold is used.—*The Therapeutic Gazette.*

---

BORIC ACID AND AFFECTIONS OF THE MOUTH.—*By A. D. Macgregor, M. B., C. M.*—In connection with the local application of boric acid in various diseased conditions of the mouth, its solubility in water and glycerine, its unirritating character, its comparatively innocuous nature, and its almost tastelessness, are greatly in its favor. More particularly is this the case in treating such conditions in children, whose oral cavities cause them so much annoyance. Speaking generally, boric acid will be found useful in all conditions of the mouth, fauces, pharynx, and nose, where there is any abrasion of the epithelium; whether it be used as a powder, gargle, mouth-wash, pigment, or confection. More definitely, I may say, it is not contradicted in any of the forms of *stomatitis*, though scarcely severe enough for the graver varieties.

In SIMPLE CATARRHAL STOMATITIS, a mouth-wash containing from 10 to 15 grains to the fluid ounce speedily cures the condition, and exercises the same beneficial influence in the *ulcerative* form, though there, in addition to the rinsing of the mouth, a local application in the form of powder or pigment should be made to the individual follicular ulcers. The powder simply consists of finely powdered boric acid, mixed in various proportions with starch: the pigments is a solution of boric acid in glycerine (1 in 4 or 5). In both cases, the addition of chlorate of potassium is advantageous; indeed, I usually combine it, but it is not essential.

For thrush in children, I especially recommend boric acid, either as a mouth-pigment or as a confection. Honey and sugar have both been condemned, as being inadmissible, in combination, for the treatment of thrush; but, so far as children are concerned, I must say I consider a confection (though made with honey), which has been impregnated with boric acid, gains more by its palatableness than it loses by tendency of the saccharine matter to further the growth of the fungus. The boric acid at once does away with this tendency. Let the pigment be frequently painted with a brush over the patches never omitting to do it after food has been taken; or, a little of the confection, simply allowed to dissolve in the mouth; and the days of the fungus will soon be ended. I have found boric acid, combined with its salt (borax), markedly beneficial. Borax alone, however, is not nearly so good.

In PHARYNGITIS AND RELAXED CONDITIONS OF THE THROAT, a gargle containing boric acid and glycerine, with either tannic acid or alum in addition, ought not to be forgotten.

In severe cases of typhoid fever the mouth is frequently hot; the lips dry, cracked, and glued to the sordes-covered teeth by inspissated mucus and saliva; the tongue dry or even glazed and hard, brown or black, crusted with a fœtid fur. Under such circumstances, a pigment, containing boric acid (30 grains), chlorate of potassium (20 grains), lemon juice (5 fluid drachms), and glycerine (3 fluid drachms), yields very comforting results. When the teeth are well rubbed with this. the sordes quickly and easily become detached; little harm will follow from the acid present. The boric acid attacks the masses of bacilli and bacteria; the chlorate of potassium cools and soothes the mucous membrane; the glycerine and lemon juice moisten the parts, and aid the salivary secretion.

A tooth powder should possess certain characteristics: it should be antiseptic, cooling, agreeable to taste and smell, and have no injurious action on the teeth. After use it should leave the teeth white, and a sensation of freshness and cleanliness in the mouth. As an antiseptic in this connection nothing can replace boric acid. For years I have used the following powder and can reccommend it:—

| | |
|---|---|
| Boric acid, finely powdered | 40 grs. |
| Chlorate of potassium | half drachm |
| Powdered guaiacum | 20 grs. |
| Prepared chalk | 1 drachm |
| Powdered carbonate of magnesia, to | 1 ounce. |
| Otto of roses | half a drop |

The boric acid in solution gets between the teeth and the edges of the gums, and there in discharges its antiseptic functions; the chlorate and guaiacum contribute their quota to the benefit of the gums and mucous membrane generally; the chalk is the insoluble powder to detach the particles of tartar which may be present; and the magnesia the more soluble soft powder which cannot harm the softest enamel.—*Dental Record from British Medical Journal.*

---

CONVULSIONS, ETC.—Convulsions may frequently be cut short, like magic, by turning the patient on his left side. The nausea as an after effect of chloroform or ether narcrosis may generally be controlled in the same manner.—*N. W. Lancet*

